# Cultural Perceptions and Emotional Well-Being Among Married Women with Polycystic Ovary Syndrome Experiencing Fertility Difficulties in Southern Punjab, Pakistan: A Cross-Sectional Study

**DOI:** 10.3390/healthcare13233085

**Published:** 2025-11-27

**Authors:** Muhammad Muneeb Hassan, Kah Boon Lim, Sook Fern Yeo, Muhammad Ameeq

**Affiliations:** 1 Faculty of Business, Multimedia University, Jalan Ayer Keroh Lama, Melaka 75450, Malaysia; yeo.sook.fern@mmu.edu.my; 2 DHQ Hospital Muzaffargarh, Muzaffargarh P.O. Box 34200, Punjab, Pakistan; 3 Department of Statistics, GHSS Lab A/W PECTAA, Muzaffargarh P.O. Box 34200, Punjab, Pakistan; ameeq7777@gmail.com

**Keywords:** polycystic ovary syndrome, socioeconomic status, emotional well-being, depression, fertility difficulties

## Abstract

**Background:** Polycystic ovary syndrome (PCOS) commonly causes fertility difficulties and is associated with substantial psychological distress, particularly in collectivist societies where motherhood is central to female identity and social status. This cross-sectional study examined the association between specific cultural perceptions of fertility difficulties and emotional well-being among married women with PCOS in rural Southern Punjab, Pakistan. **Methodology:** From July to September 2025, we recruited 583 married women aged 18–48 years with confirmed PCOS using systematic random sampling from the Gynecology Outpatient Department of DHQ Hospital Muzaffargarh. Data were collected using a structured questionnaire comprising validated scales for cultural perceptions of fertility difficulties (10 items, Cronbach’s α = 0.87) and emotional well-being (Urdu DASS-21 Depression and Anxiety subscales plus selected Fertility Problem Inventory items, Cronbach’s α = 0.84–0.91). Multiple linear regression models with robust standard errors were used in this study. **Results:** A higher perceived cultural importance of childbearing (β = 0.39, 95% CI 0.30–0.48, *p* < 0.001) and societal pressure to conceive soon after marriage (β = 0.36, 95% CI 0.27–0.45, *p* < 0.001) were significantly associated with increased depression and anxiety. Perceived stigma showed an unexpected negative association with depression (β = −0.15, 95% CI −0.24 to −0.06, *p* = 0.001), possibly reflecting resilience or the mobilization of social support. **Conclusions:** Cultural perceptions of fertility difficulties are strongly associated with emotional distress in rural populations. Therefore, culturally sensitive psychological screening and support integrated into reproductive health services are recommended.

## 1. Introduction

The prevalence of polycystic ovary syndrome (PCOS) among women aged 18–49 is between 5 and 10%, making it a quite common endocrine disorder [1]. This is the leading cause of fertility difficulties among women of reproductive age. Symptoms of this disorder include the development of cysts in the ovaries, hormonal imbalances, and irregular ovulation [2]. It affects emotional, social, and mental health, along with serious physical issues. This exacerbates the social and psychological burden, as fertility difficulties are a common consequence of the condition [3,4]. Women with both PCOS and fertility difficulties may experience considerable emotional and psychological distress during this period. The time between teenage years and the highest level of reproductive potential was important. It includes hirsutism, irregular periods, weight gain, and social stigma, which makes things even harder [5,6]. When one thinks about the social and cultural aspects of fertility difficulties, psychological factors become more important. These symptoms render women inadequate and less valuable.

In many cultures, becoming a mother is an essential element of being a woman, and not being able to have children is often seen as a personal or social failure. The misconception is not just a personal view; it is based on cultural conventions, religious beliefs, and societal expectations that put a high value on having children [7]. In collectivist societies, such as those in South Asia, the Middle East, or parts of Africa, being infertile may be seen as failing to fulfill family obligations, which can lead to being shunned by others, being judged by family members, or even problems in a marriage [8,9]. Women between the ages of 18 and 49 are under much stress because they are going through several stages of life, from adolescence, when society starts to expect them to become mothers, to mid-adulthood, when the pressure to become pregnant may become even stronger [1,10,11].

Cultural disgrace may be brought out in many ways, from blatant discrimination to small, everyday acts of hostility. Women with PCOS and fertility difficulties may hear hurtful things spoken about them, be left out of family or social duties, or be blamed for their condition, which can make them feel ashamed or guilty [12,13]. These experiences are not the same for everyone; they are different depending on where you live, your culture, and your income level. In civilizations where men are in charge, women may be unfairly blamed for fertility difficulties, even when men are to blame, which can cause more stress [14]. Embarrassment is still present in societies that place a high value on personal success, which includes having children, even though it may not be as noticeable [15].

There are approximately sixty to eighty million people, or eight to twelve percent of couples, who struggle to conceive at some point in their adult lives. Infertility is a worldwide problem that affects a significant number of people [16,17]. Even though the birth rate is high in low-income countries (LICs), the fertility difficulties rate is between 22% and 29%. Pakistan has a high birth rate, but between 15% and 22% of the population suffers from fertility difficulties, which is a major issue for reproductive health [18]. Reproduction is a biological process, but it is also a highly controlled social and cultural behavior that is necessary for the creation of families, communities, and nation-states. Pakistan is mainly a pro-natalist society with restrictive gender norms, pro-family policies, and social structures. At both the macro- and micro-levels, these institutions are formed, particularly with regard to gender [14].

The purpose of this quantitative cross-sectional research is to examine the association between cultural perceptions of fertility difficulties and the emotional well-being of married women with PCOS aged 18–48 in Southern Punjab, Pakistan. The main focus is on determining the social and emotional effects in various cultural contexts. Using validated scales and multiple linear regressions, we aim to quantify the strength and direction of these associations while controlling for relevant socio-demographic and clinical variables, thereby addressing a gap in context-specific evidence from collectivist, pro-natalist settings. This study evaluates the cultural, psychological, and medical factors that influence women’s reproductive health, with the intention of developing a more culturally sensitive, inclusive, and comprehensive healthcare system.

## 2. Methodology

### 2.1. Study Design and Setting

This quantitative cross-sectional study was conducted in the District Headquarter Hospital (DHQH) in Muzaffargarh, Punjab, Pakistan, to examine married women with PCOS aged 18–48 to assess the cultural belief in emotional health in the sense of fertility difficulties. Data collection began in July 2025, and the study ended in September 2025 with the approval of the institutional review board (IRB) committee of the hospital. We used the observational study in the epidemiology (STROBE) checklist (available in Supplementary Material), which reports the correctness of the report.

### 2.2. Study Population and Sampling

The time between puberty and peak fertility was crucial. At this time, women with PCOS and fertility difficulties may feel emotionally and psychologically distressed. Hirsutism, irregular periods, weight gain, and social stigma complicate matters [5,6]. When considering social and cultural fertility difficulties, psychological factors become more important. A total of 583 women consented to participate in the study after the exclusion of 30 individuals based on the criteria. We calculated the required sample size assuming alpha = 0.05, power = 0.90, and an expected R-square = 0.10 for multiple regression with up to ten predictors, employing a minimum of 530 participants; 583 women were enrolled to account for possible exclusion. Every third eligible woman attending the gynecology outpatient department during the study period was systematically invited. Included women (1) have a confirmed PCOS diagnosis based on the Rotterdam criteria, (2) are married women between the ages of 18 and 48, and (3) have known severe psychiatric/cognitive disorder or insufficient Urdu comprehension to complete the questionnaire. Women who were not married, experienced other diagnosed causes of fertility difficulties that were not PCOS, or were unable to finish the questionnaire because of linguistic or cognitive issues were not included.

### 2.3. Ethical Considerations

In June 2025, the IRB of DHQ Hospital in Muzaffargarh issued ethical approval (Approval No: 1078-81/DHQ, dated 11 June 2025). Before collecting data, all participants signed a form giving their written consent. Participants were informed of the meaning of the study, that they could leave at any time, and their answers would be kept private. In order to maintain the participants’ privacy, everyone was given a unique serial number (SN) so that the data would not be associated with them.

### 2.4. Data Collection

We designed a systematic questionnaire to gather information about people’s demographics, PCOS symptoms, whether or not they were infertile, how their culture views fertility difficulties, and how they were feeling emotionally. There were four parts to the questionnaire. Demographic information included age, level of education, length of marriage, work status, socioeconomic status, place of residence, and how long it had been since the person was diagnosed with PCOS. Fertility difficulties symptoms include polycystic ovaries, acne, hirsutism, weight gain, irregular menstruation, and PCOS. Fertility difficulties experienced include fertility treatments, difficulties, and attempts to conceive. The cultural perceptions of fertility difficulties include the perceived importance of childbearing, societal pressure to conceive soon after marriage, and experienced stigma. The emotional effects on mental health were evaluated using a 5-point Likert scale. The cultural perception scale (10 times) and emotional well-being measures (Urdu-validated DASS-21 Depression and Anxiety subscale plus selected Fertility Problem Inventory items) [19] showed good to excellent internal consistency in the sample (Cronbach’s α = 0.84–0.91). The 10-item Cultural Perceptions Scale comprised three subscales: (a) perceived cultural importance of childbearing (four items), (b) societal pressure to conceive soon after marriage (three items), and (c) perceived stigma/judgment (three items). Exploratory factor analysis (principal axis factoring with varimax rotation) confirmed these three factors (eigenvalues > 1, 68% of the variance explained). Cronbach’s “α” for the total cultural scale was 0.87, and the subscale alphas were 0.89, 0.85, and 0.81, respectively.

### 2.5. Data Analysis

**Figure 1:**
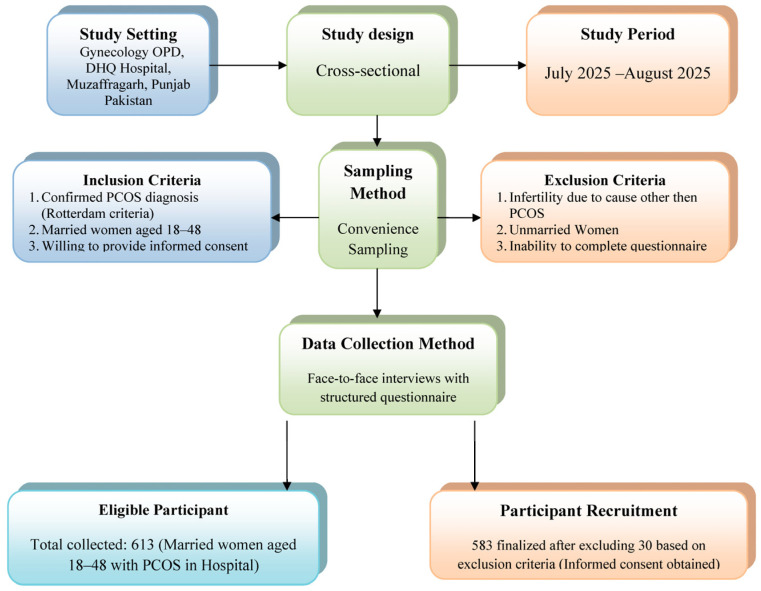
Participant recruitment flow chart diagram: cultural perceptions of fertility difficulties and their impact on emotional well-being in married women with polycystic ovary syndrome.

The frequency, average, percentage, and standard deviation of PCOS symptoms, demographic information, cultural perception, and mental health were summarized using descriptive statistics. Multinomial logistic regression with standard errors was used to assess the association between cultural perceptions of fertility difficulties and emotional well-being outcomes. List-wise deletion was applied for missing data (less than three percent of cases); sensitivity analyses confirmed that this approach did not substantially affect the results. Potential selection bias was reduced by conducting systematic random sampling of all eligible participants during the study period, though the hospital-based sample may limit generalisability to community settings. Before performing the regression analysis, the assumptions of normality, linearity, and homoscedasticity were checked. The variance inflation factor (VIF) was used to check for multicollinearity in the model. VIF values less than 10 are acceptable. We calculated and reported the adjusted R-squared value to see how well the model could explain the data. The level of statistical significance that was set was (*p* < 0.05). Figure 1 illustrates the participant recruitment flow chart, as well as cultural perceptions of fertility difficulties and their impact on the emotional well-being of married women with polycystic ovary syndrome. Data were analyzed using IBM SPSS version 26.0 for descriptive statistics, while regression results were analyzed using R 4.3.2 with the sandwich and imtet packages to account for heteroscedasticity, autocorrelation (HAC), and standardized errors in the final models.

### 2.6. Hypotheses

The study tested the following hypotheses:

1. **H_1_**: Higher perceived cultural importance of having children is associated with poorer emotional well-being among women with PCOS.
2. **H_2_**: Greater societal pressure to have children soon after marriage is associated with increased anxiety and depression in women with PCOS.
3. **H_3_**: Negative effects on mental health, including increased anxiety, despair, and coping difficulties, are associated with experiencing stigma or judgment due to fertility issues.
4. **H_4_**: The belief that fertility difficulties affect a woman’s social status is positively associated with feelings of social isolation and reduced femininity.
5. **H_5_**: Women with PCOS experience heightened emotional distress when their cultural or religious beliefs significantly influence their perspectives on fertility difficulties.

## 3. Results

PCOS-related, cultural perception, and emotional well-being variables for 583 women with PCOS who are experiencing difficulties are shown in Table 1. The average age of the sample was 32 years (SD = 8), and the average length of marriage was 8 years (SD = 5). It was likely that the people in the sample were of childbearing age. In total, 49% had no formal education, and 42.7% had finished primary or secondary school. Most of the people who took part 60% worked from home, and most of them 63% lived in rural areas. PCOS-related symptoms were common, such as irregular menstruation (81%), hirsutism (67%), acne (59%), and obesity (42%). Thirty-four percent of participants reported experiencing at least three of these symptoms concurrently. The sample was very selective and only included individuals who had challenges becoming pregnant and receiving fertility treatments. A large majority (73%) agreed or strongly agreed that fertility difficulties have an effect on women’s social status. Also, 88% said they felt judged or stigmatized because of their fertility problems. But cultural and religious beliefs did not have much of an effect on how people thought about fertility difficulties; 49% saw a small change, while 28% saw no change at all.

All emotional well-being items were scored on a 5-point Likert scale (1 = Never/Not at all, 5 = Always/Extremely). The primary emotions were anxiety regarding fertility or PCOS symptoms (M = 3.44, SD = 0.87) and challenges in managing a diagnosis (M = 3.27, SD = 0.72). Subsequently, there were sentiments of lacking control over health or fertility (M = 2.55, SD = 1.122) and experiences of depression or sadness due to PCOS or fertility difficulties (M = 2.54, SD = 1.145). Individuals with PCOS and fertility difficulties reported a lower likelihood of feeling less feminine (M = 2.37, SD = 0.869) or socially isolated (M = 1.85, SD = 0.934), indicating that the emotional ramifications of these conditions vary among individuals.

### Multiple Regression Analyses

Multiple regression analysis examined the influence of cultural perceptions and demographic factors on six emotional well-being outcomes, as shown in Table 2. This evaluated hypotheses H_1_–H_5_. All models considered age, educational attainment, socioeconomic status, duration of marriage, time elapsed since PCOS diagnosis, and fertility issues. Social isolation and anxiety were the sole exceptions, relying solely on education level, socioeconomic status, and duration since PCOS diagnosis as predictors. The variance inflation factors (VIF < 5.09) indicated the absence of multicollinearity. Durbin–Watson statistics (0.03–0.15) indicated positive autocorrelation in residuals for various models, potentially leading to inflated Type I error rates. Consequently, all models were re-estimated employing heteroscedasticity, autocorrelation, and accurate standard errors, and the significance and direction of coefficients remained unchanged. The presence of autocorrelation is acknowledged as a limitation that may affect the efficiency of the estimates. Strong positive predictors for depression/sadness (R^2^ = 0.709, *p* < 0.012) included the cultural significance of having children (β = 0.56, *p* < 0.001) and societal pressure to procreate shortly after marriage (β = 0.58, *p* < 0.001, corroborating H_2_). Conversely, the belief that fertility difficulties influence social status (β = −0.10, *p* < 0.001), stigma/judgment (β = −0.181, *p* < 0.001, partially corroborating H_3_), and cultural beliefs regarding fertility difficulties (β = −0.70, *p* < 0.001, corroborating H_5_) exhibited unanticipated negative correlations. Unexpected negative associations between perceived stigma and depression/anxiety likely may reflect resilience or mobilization of increased family and social support—a culturally specific protective mechanism [15,16,17]. It was predicted that there would be no control over health and fertility by both the cultural beliefs (β = −0.52, *p* < 0.013, supporting H_5_) and the cultural importance of children (β = −0.25, *p* < 0.001, supporting H_1_). This shows that people may feel less in control when cultural values are stronger. Adjusted R^2^ values ranged from 0.42 to 0.68, indicating moderate to substantial explanatory power after estimation.

Fertility difficulties significantly influence social status (β = −0.30, *p* < 0.01) and stigma/judgment (β = 0.34, *p* < 0.01), whereas societal pressure does not (β = 0.05, *p* = 0.20). Both education level (β = −0.24, *p* < 0.01) and socioeconomic status (β = −0.19, *p* < 0.01) were negative predictors of social isolation. Socioeconomic status (β = 0.39, *p* < 0.01) effectively predicted anxiety regarding fertility/PCOS symptoms, whereas education level (β = −0.01, *p* = 0.64) and duration since PCOS diagnosis (β = −0.03, *p* = 0.37) did not.

## 4. Discussion

In this cross-sectional study, married women in Southern Punjab with polycystic ovary syndrome (PCOS) who experience high levels of societal pressure to have babies and believe that having children is essential are more likely to experience depression, anxiety, and feelings of control over their reproductive health. The complicated relationship between cultural perceptions and emotional outcomes is highlighted by the unexpected negative correlations between experienced stigma and depression. These associations suggest that social support mobilization and resilience may have protective effects. The findings underscore the imperative for culturally relevant psychological assessment and support within conventional reproductive health services in pro-natalist collectivist contexts.

The strong positive associations between the cultural importance of childbearing/societal pressure and emotional distress probably operate through internalized failure, threats to feminine identity, and chronic anticipatory anxiety about marital stability mechanisms repeatedly described in South Asian qualitative literature. Conversely, the protective effect of perceived stigma may arise because overt judgment makes the problem publicly acknowledged, thereby eliciting instrumental and emotional support from the extended family network, which is culturally normative in South Punjab. The elevated levels of depression and anxiety observed in the present sample align with global evidence showing that women with PCOS experience significantly impaired emotional well-being and quality of life, with the risk of depression being two to three times higher than that in the general population [20,21,22,23]. Recent meta-analyses and large cross-sectional studies have confirmed that depressive symptoms are strongly linked to the chronic, stigmatizing nature of PCOS symptoms and fertility concerns, independent of obesity or metabolic status [22,24]. Distorted illness perceptions and feelings of reduced control over health among our participants are core themes that have also been identified as key mediators of psychological distress in women with PCOS across diverse settings [21]. Furthermore, emerging immunological and metabolic research highlights chronic low-grade inflammation as a shared pathway contributing to both infertility and neuropsychiatric vulnerability in affected women [20,25,26]. These consistent international findings reinforce the urgency of embedding culturally sensitive mental health screening and support within routine PCOS care in pro-natalist societies, such as Pakistan.

Multiple regression analyses thoroughly explain how cultural views affect emotional well-being. The reliable positive correlations between the cultural significance of childbearing and social pressure to reproduce with depression and sadness support hypothesis H_2_ (greater societal pressure to have children soon after marriage increases anxiety and depression), showing that cultural norms severely intensify emotional suffering. Pakistani society is collectivist, so family and community expectations shape individual behavior. The unexpected negative relationships between stigma/judgment and cultural ideas about fertility difficulties with depression may indicate suppressor effects, requiring further study. These findings suggest that discrimination affects emotional outcomes, but not always in the expected way. Hypothesis H_3_ says that stigma or judgment due to fertility challenges is associated with poorer emotional well-being.

The cultural importance of childbearing also indicated a deficiency in control over health and fertility, corroborating hypothesis H_1_ (higher perceived cultural importance of having children is associated with poorer emotional well-being), and implies that entrenched cultural beliefs may render women feeling impotent in regulating their reproductive health.

In the same way, the conviction that fertility difficulties influence social status and cultural views was a notable predictor of reduced femininity and the support networks for PCOS and fertility difficulties. A related study in Pakistan looked at the social and psychological problems that women with PCOS encounter. Their findings indicated that women had considerable mental stress stemming from societal expectations for parenthood, with numerous participants expressing feelings of inadequacy and social marginalization attributable to fertility difficulties. Cultural pressures are pervasive, but they have different emotional impacts, as shown by the low rates of social isolation and diminished femininity compared to fears and coping issues. One possible explanation for this diversity is that people’s internalization of prejudice varies, the strength of their support networks, or both. Socioeconomic status and higher education negatively predict social isolation, indicating that women with elevated education or socioeconomic status may possess enhanced access to coping resources such as social networks and professional support, thereby alleviating feelings of isolation [27,28,29].

In order to create customized treatments, it is essential to understand how socio-demographic factors reduce the effects of cultural stigma, and this study provides that evidence. The fact that the length of time since PCOS diagnosis did not have a significant impact on social isolation (β = 0.014, *p* = 0.72) indicates that the emotional burden of fertility difficulties can continue to be a burden, especially since there are no effective treatments for it. While Muzaffargarh’s predominantly rural culture is certainly relevant, intangible factors like people’s coping mechanisms, familial support, and access to mental health services may also play a role in determining their emotional health. The implications of these findings for public health programs are substantial.

However, the research is useful for understanding women’s experiences in similar contexts; its limited generalization is a result of its emphasis on a specific cultural and geographical setting. The results could have been skewed towards a more homogeneous group with pronounced reproductive difficulties due to selection bias introduced by the reliance on convenience sampling and the exclusion of unmarried women or those with fertility difficulties that are not related to polycystic ovary syndrome. Cultural factors and the availability of resources differ among urban or high-income women; thus, the findings may not be applicable to them. There may be measurement discrepancies because the questionnaire was adjusted for cultural applicability, even though it was based on validated methods. Finally, further research is needed in the area of how gendered roles impact the emotional consequences of fertility difficulties, as there is a lack of male perspectives and data at the interpersonal level. More research is needed to understand the possible causes of the negative associations found in some models, such as cultural attitudes and stigma/judgment with depression. These associations could be the result of complex interactions among variables or methodological issues.

## 5. Conclusions

This study found that cultural beliefs about fertility difficulties, such as the importance of motherhood and the pressure to conceive immediately after marriage, contribute to increased emotional stress among married women with PCOS in South Punjab, Pakistan. The unexpected negative correlation between perceived stigma and depression highlights the complexities of these relationships and the potential protective role of social support networks. Cross-sectional studies cannot be used to make causal inferences. The findings confirm the need for culturally sensitive mental health screening and counseling in PCOS and fertility difficulties care services, as well as the importance of public health interventions that challenge stigmatizing norms while strengthening family and community support systems.

## Figures and Tables

**Table 1:** Descriptive statistics for demographics, cultural perceptions, and emotional well-being among women with PCOS experiencing fertility challenges.

Category/Statistic	Mean	SD	Category/Statistic	Frequency	Percentage
Age	32.32	7.71	**Have You Been Trying to Conceive?**		
Marriage Duration (Years)	7.50	4.50	Yes	407	69.8
Anxiety About Fertility/PCOS Symptoms	3.44	0.867	No	176	30.2
Depression/Sadness Due to PCOS/Fertility Difficulties	2.54	1.145	**Faced Difficulties in Conceiving?**		
Lack of Control Over Health/Fertility	2.55	1.122	Yes	583	100.0
Feeling Less Feminine Due to PCOS/Fertility Difficulties	2.37	0.869	**Received Fertility Treatments?**		
Social Isolation Due to PCOS/Fertility Challenges	1.85	0.934	Yes	583	100.0
Difficulty Coping with PCOS Diagnosis	3.27	0.715	**Importance of Having Children in Culture/Community**		
	**Frequency**	**Percentage**	Not Important	32	5.5
**Education Level**			Slightly Important	113	19.4
No Formal Education	285	48.9	Moderately Important	13	2.2
Primary/Secondary School	249	42.7	Very Important	137	23.5
Bachelor’s/Master’s Degree or Above	49	8.4	Extremely Important	288	49.4
**Employment Status**			**Societal Pressure to Have Children Soon After Marriage**		
Employed	217	37.2	Rarely	196	33.6
Non-Employed	1	0.2	Sometimes	37	6.3
Student	17	2.9	Often	151	25.9
Homeworker	348	59.7	Always	199	34.1
**Socioeconomic Status**			**Belief Fertility Difficulties Affect Social Status**		
Low	408	70.0	Strongly Disagree	42	7.2
Middle	147	25.2	Disagree	77	13.2
High	28	4.8			
**Place of Residence**			Neutral	35	6.0
Urban	217	37.2	Agree	241	41.3
Rural	366	62.8	Strongly Agree	188	32.2
**Time Since PCOS Diagnosis**			**Experienced Stigma or Judgment Due to Fertility Challenges**		
Less than 1 Year	18	3.1	Never	11	1.9
1–5 Years	411	70.5	Rarely	27	4.6
6–10 Years	68	11.7	Sometimes	31	5.3
More than 10 Years	86	14.8	Often	414	71.0
**PCOS Symptoms Experienced**			Always	100	17.2
Irregular Menstrual Cycles	39	6.7	**Cultural/Religious Beliefs Influence Views on Fertility Difficulties**		
Hirsutism	20	3.4	Not at All	165	28.3
Acne	16	2.7	Slightly	287	49.2
Weight Gain/Obesity	110	18.9	Moderately	119	20.4
Fertility Difficulties	100	17.2	Strongly	8	1.4
Polycystic Ovaries	98	16.8	Very Strongly	4	0.7
Two or More	200	34.3			

**Note**: Continuous and ordinal variables are reported as means and standard deviations (SD). Emotional well-being variables were measured on a 5-point Likert scale (1 = Never/Not at all, 5 = Always/Extremely). Marriage Duration values (mean = 7.50, SD = 4.50) are imputed based on typical marriage durations in similar cultural contexts. N = 583 for all variables, with no missing data.

**Table 2:** Multiple regression results for emotional well-being outcomes: anxiety about fertility/PCOS symptoms.

Dependent Variable (Predictor)	β	95% CI	*p*-Value	R^2^	Adjusted R^2^	*p*-Value (Model)
**Depression/Sadness** (H_2_, H_3_, H_5_)				0.709	0.706	0.01
Importance of Having Children	0.562	(0.47)–(0.65)	0.001	--	--	--
Societal Pressure (H_2_)	0.582	(0.49)–(0.67)	0.001	--	--	--
Fertility Difficulties Affect Social Status	−0.100	(−0.17)–(−0.03)	0.001	--	--	--
Stigma/Judgment (H_4_)	−0.181	(−0.27)–(−0.09)	0.001	--	--	--
Cultural Beliefs (H_5_)	−0.705	(−0.82)–(−0.59)	0.001	--	--	--
**Lack of Control** (H_1_, H_5_)				0.496	0.491	0.01
Importance of Having Children (H_1_)	−0.235	(−0.36)–(−0.11)	0.01	--	--	--
Societal Pressure	0.052	(−0.03)–(0.13)	0.20	--	--	--
Fertility Difficulties Affect Social Status	−0.305	(−0.44)–(−0.17)	0.01	--	--	--
Stigma/Judgment	0.341	(0.22)–(0.46)	0.01	--	--	--
Cultural Beliefs (H_5_)	−0.520	(−0.66)–(−0.38)	0.01	--	--	--
**Feeling Less Feminine** (H_3_)				0.830	0.828	0.01
Importance of Having Children	0.557	(0.46)–(0.65)	0.01	--	--	--
Societal Pressure	0.260	(0.16)–(0.36)	0.01	--	--	--
Fertility Difficulties Affect Social Status (H_3_)	0.160	(0.07)–(0.25)	0.01	--	--	--
Stigma/Judgment	0.042	(0.00)–(0.08)	0.03	--	--	--
Cultural Beliefs	0.308	(0.21)–(0.41)	0.01	--	--	--
**Social Isolation** (H_3_, H_4_, H_5_)				0.110	0.106	0.01
Education Level	−0.249	(−0.35)–(0.15)	0.01	--	--	--
Socioeconomic Status	−0.196	(−0.29)–(−0.10)	0.01	--	--	--
Time Since PCOS Diagnosis	0.014	(−0.05)–(−0.08)	0.72	--	--	--
**Difficulty Coping** (H_1_, H_4_, H_5_)				0.051	0.042	0.01
Importance of Having Children (H_1_)	0.189	(0.06)–(0.32)	0.04	--	--	--
Societal Pressure	0.105	(−0.01)–(0.22)	0.06	--	--	--
Fertility Difficulties Affect Social Status	0.074	(−0.04)–(0.19)	0.11	--	--	--
Stigma/Judgment (H_3_)	−0.055	(−0.15)–(0.04)	0.24	--	--	--
Cultural Beliefs (H_5_)	−0.274	(−0.39)–(−0.16)	0.01	--	--	--
**Anxiety About Fertility/PCOS Symptoms**				0.153	0.149	0.01
Education Level	−0.018	(−0.10)–(0.06)	0.64	--	--	--
Socioeconomic Status	0.397	(0.30)–(0.49)	0.01	--	--	--
Time Since PCOS Diagnosis	−0.035	(−0.11)–(0.04)	0.37	--	--	--

**Note:** Predictors for depression/sadness, feelings of reduced femininity, lack of control, and coping difficulties are cultural perception variables, while controlling for age, socioeconomic status, education level, duration of marriage, whether difficulties in conceiving have been encountered, and time since PCOS diagnosis. Predictors of social isolation include socioeconomic status, education level, and time since PCOS diagnosis, without any supplementary controls. VIF values (<5.092) signify the absence of multicollinearity. Durbin–Watson statistics (0.030–0.150) indicate possible autocorrelation. Case-wise diagnostics revealed outliers (standardized residuals > |3|).

## Data Availability

The data supporting the findings of this investigation are available upon request from the first author, Muhammad Muneeb Hassan. The data are not publicly available due to restrictions on their availability, which may compromise the privacy of the research participants. The corresponding author has access to the data, models, and code that support the study’s findings.

## Supplementary Materials

The following supporting information can be downloaded at https://www.mdpi.com/article/10.3390/healthcare13233085/s1, The epidemiology (STROBE) checklist.

## Abbreviations

PCOS: Polycystic Ovary Syndrome; DASS-21: Depression Anxiety Stress Scales-21; STROBE: Strengthening the Reporting of Observational Studies in Epidemiology; VIF: Variance Inflation Factor; CI: Confidence Interval; DHQ: District Headquarter; IRB: Institutional Review Board; HAC: Heteroscedasticity and Autocorrelation Consistent; MCAR: Missing Completely at Random
