# Supplementary material for: Cultural Perceptions and Emotional Well-Being Among Married Women with Polycystic Ovary Syndrome Experiencing Fertility Difficulties in Southern Punjab, Pakistan: A Cross-Sectional Study
Source: Healthcare (Basel). 2025 Nov 27;13(23):3085. doi: 10.3390/healthcare13233085 (PMC12692285; doi:10.3390/healthcare13233085)
Supplement: Supplementary file 1 [file healthcare-13-03085-s001.zip › healthcare-3997793-supplementary.pdf]

STROBE Statement—checklist of items that should be included in reports of cross-sectional studies

	Item No	Recommendation	Location in the manuscript (Page / Section)
**Title and abstract**	1	(*a*) Indicate the study's design with a commonly used term in the title or the abstract	Title & Abstract (first page)
		(*b*) Provide in the abstract an informative and balanced summary of what was done and what was found	Abstract (first page)
**Introduction**			
Background/rationale	2	Explain the scientific background and rationale for the investigation being reported	Introduction (pp. 2-4)
Objectives	3	State specific objectives, including any prespecified hypotheses	End of Introduction (p. 4) and Section 2.6 Hypotheses
**Methods**			
Study design	4	Present key elements of study design early in the paper	Methods → 2.1 Study Design and Setting (p. 4)
Setting	5	Describe the setting, locations, and relevant dates, including periods of recruitment, exposure, follow-up, and data collection	2.1 Study Design and Setting (p. 4)
Participants	6	(*a*) *Cohort study*—Give the eligibility criteria, and the sources and methods of selection of participants. Describe methods of follow-up<br>*Case-control study*—Give the eligibility criteria, and the sources and methods of case ascertainment and control selection. Give the rationale for the choice of cases and controls<br>*Cross-sectional study*—Give the eligibility criteria, and the sources and methods of selection of participants	2.2 Study Population and Sampling (pp. 4-5)
		(*b*) *Cohort study*—For matched studies, give matching criteria and number of exposed and unexposed<br>*Case-control study*—For matched studies, give matching criteria and the number of controls per case	NA
Variables	7	Clearly define all outcomes, exposures, predictors, potential confounders, and effect modifiers. Give diagnostic criteria, if applicable	2.4 Data Collection & 2.5 Data Analysis (pp. 5-7)
Data sources/ measurement	8*	For each variable of interest, give sources of data and details of methods of assessment (measurement). Describe comparability of assessment methods if there is more than one group	2.4 Data Collection (pp. 5-6)
Bias	9	Describe any efforts to address potential sources of bias	2.5 Data Analysis (p. 6) and Discussion → Limitations (pp. 13-14)
Study size	10	Explain how the study size was arrived at	2.2 Study Population and Sampling (p. 5)
Quantitative variables	11	Explain how quantitative variables were handled in the analyses. If applicable, describe which groupings were chosen and why	2.5 Data Analysis (p. 6)
Statistical methods	12	(*a*) Describe all statistical methods, including those used to control for confounding	2.5 Data Analysis (pp. 6-7)
		(*b*) Describe any methods used to examine subgroups and interactions	Not performed
		(*c*) Explain how missing data were addressed	2.5 Data Analysis (p. 6)
		(*d*) *Cohort study*—If applicable, explain how loss to follow-up was addressed<br>*Case-control study*—If applicable, explain how matching of cases and controls was addressed	NA

		Cross-sectional study—If applicable, describe analytical methods taking account of sampling strategy	
		(*e*) Describe any sensitivity analyses	NA

Continued on next page

**Results**			
Participants	13*	(a) Report numbers of individuals at each stage of study—eg numbers potentially eligible, examined for eligibility, confirmed eligible, included in the study, completing follow-up, and analysed	Figure 1 (referenced p. 7) and text in Results (p. 8)
		(b) Give reasons for non-participation at each stage	30 excluded based on criteria (2.2)
		(c) Consider use of a flow diagram	Figure 1 (p. 7)
Descriptive data	14*	(a) Give characteristics of study participants (eg demographic, clinical, social) and information on exposures and potential confounders	Results → Table 1 (pp. 8-9)
		(b) Indicate number of participants with missing data for each variable of interest	2.5 (p. 6) and Table 1 note
		(c) *Cohort study*—Summarise follow-up time (eg, average and total amount)	NA
Outcome data	15*	*Cohort study*—Report numbers of outcome events or summary measures over time	NA
		Case-control study—Report numbers in each exposure category, or summary measures of exposure	NA
		Cross-sectional study—Report numbers of outcome events or summary measures	Results → Table 1 & Table 2 (pp. 8-11)
Main results	16	(*a*) Give unadjusted estimates and, if applicable, confounder-adjusted estimates and their precision (eg, 95% confidence interval). Make clear which confounders were adjusted for and why they were included	Table 2 and text in Results (pp. 9-11)
		(*b*) Report category boundaries when continuous variables were categorized	Not categorized
		(*c*) If relevant, consider translating estimates of relative risk into absolute risk for a meaningful time period	NA
Other analyses	17	Report other analyses done—eg analyses of subgroups and interactions, and sensitivity analyses	None beyond main models and sensitivity checks
**Discussion**			
Key results	18	Summarise key results with reference to study objectives	Discussion → first paragraph & Conclusion (pp. 12-15)
Limitations	19	Discuss limitations of the study, taking into account sources of potential bias or imprecision. Discuss both direction and magnitude of any potential bias	Discussion → penultimate paragraph (pp. 13-14)
Interpretation	20	Give a cautious overall interpretation of results considering objectives, limitations, multiplicity of analyses, results from similar studies, and other relevant evidence	Discussion & Conclusion (pp. 12-15)
Generalisability	21	Discuss the generalisability (external validity) of the study results	Discussion → Limitations (p. 14)
**Other information**			
Funding	22	Give the source of funding and the role of the funders for the present study and, if applicable, for the original study on which the present article is based	Funding section (p. 15)

*Give information separately for cases and controls in case-control studies and, if applicable, for exposed and unexposed groups in cohort and cross-sectional studies.

Note: An Explanation and Elaboration article discusses each checklist item and gives methodological background and published examples of transparent reporting. The STROBE checklist is best used in conjunction with this article (freely available on the Web sites of PLoS Medicine at http://www.plosmedicine.org/, Annals of Internal Medicine at http://www.annals.org/, and Epidemiology at http://www.epidem.com/). Information on the STROBE Initiative is available at www.strobe-statement.org.
